# Anesthetic management of a patient with localised scleroderma

**DOI:** 10.1186/s40064-016-3189-y

**Published:** 2016-09-07

**Authors:** Fangfan Ye, Gaoyin Kong, Jia Huang

**Affiliations:** 1 Department of Anesthesiology, Hunan Provincial People’s Hospital, The First Affiliated Hospital of Hunan Normal University, JieFang Road West 61, Changsha, Hunan Province People’s Republic of China; 2 Department of Intensive Care Unit, The Second Xiangya Hospital, Central South University, Renmin Road 139, Changsha, Hunan Province People’s Republic of China

**Keywords:** Anesthetic management, Scleroderma, Shikani

## Abstract

**Introduction:**

Scleroderma is a progressive fibrotic disorder of connective tissue which can present multiple anesthetic challenges to anesthetists, especially airway management. Awake intubation with fiberoptic bronchoscope is widely accepted and implemented for progressive systematic scleroderma patients. With the development and improvement of intubation devices these years, there is no report addressing other intubation methods for sclerotic patients.

**Case description:**

A 47 year-old, 42-kg man with 1-year history of localized scleroderma was scheduled for the operation of inner fixation after 6 days of his acetabular fracture. With careful pre-anesthesia assessment, we chose general anesthesia and intubated the patient with Shikani optical stylet under rapid sequence introduction successfully.

**Discussion:**

Scleroderma is a multisystem disease that can affect every aspect of anesthesia especially airway management, which can pose a significant challenge for anesthesiologists. As a result, understanding its pathophysiological changes and implementing a meticulous pre-anesthesia check-up are essential when making an anesthetic plan.

**Conclusion:**

Anesthetists should have a thorough consideration of all the patho-physiological changes in patients with scleroderma, so as to make a full preparation peri-operatively. Shikani optical stylet may become an alternation for difficult airway in patients with scleroderma.

## Background

Scleroderma is a progressive fibrotic disorder of connective tissue that can involve skin, airway, musculoskeletal, gastrointestinal, cardiopulmonary and renal system, which can present multiple anesthetic challenges to anesthetists. As a result, potential risks and benefits should be anticipated carefully. In this article, we present a patient with acetabular fracture complicated with localised scleroderma.

## Case report

A 47 year-old, 42-kg man with 1-year history of localised scleroderma referred to Trauma Department in our hospital because of acetabular fracture due to a car accident. After a series of pre-operation examinations, he was scheduled for a selective operation of inner fixation 6 days after his trauma. During the preanesthesia assessment, we found him not on any drugs but some kind of Chinese medication occasionally for scleroderma for about a year because of the side effects of the Chinese medications. There was no history of any surgery or anesthesia. Examination of his airway revealed microstomia with mouth opening of 2.5 cm and a Mallampati class III airway. However, there was no problem with motion of his head and neck, as the skin on his neck was not affected by scleroderma. On general physical examination, vital signs were stable, heart sounds were normal and breath sounds were clear bilaterally. His face was smooth and wrinkleless, his forearms had non-pitting edema, and his fingers were shortened and sausage-like, with flexion contractures on both wrists and fingers. Laboratory tests of total blood cell count, arterial blood gas analysis, pulmonary, liver and kidney function, ECG, echocardiography and X-ray were taken, all of which were normal except for mild anemia (Hb 90 g/L), which could be inferred that his viscera were not badly affected by scleroderma. For the reason of his anemia and predicted volume of blood loss he might have during the surgery, 2 U of packed red blood cell was prepared and ready for use on the surgery day.

In the operating room, standard monitors were attached, showing heart rate of 72 bpm and blood pressure of 112/65 mmHg. However, SpO_2_ could not be read on any of his fingers. We assumed it may because of skin thickening and vasospasm on his hands and fingers, so we moved the probe to his toes and finally got the value of 95 % while he was breathing room air. Peripheral intravenous access was obtained with difficulty hence awake right internal jugular venous insertion was achieved under regional anesthesia. Ranitidine 50 mg and tropisetron 3 mg were given intravenously. An ENT surgeon for tracheostomy was kept ready. After pre-oxygenation, anesthesia was induced with penehyclidine hydrocloride 0.2 mg, midazolam 2 mg, sufentanyl 25 μg, propofol 40 mg. After ensuring optimal bag and mask ventilation, neuromuscular blockade was administered with 60 mg succinylcholine to facilitate tracheal intubation. With epiglottis, glottis and tracheal cartilage clearly seen under Shikani Optical Stylet (SOS), endotracheal tube of ID (internal diameter) 7 mm was achieved and confirmed by symmetrical breath sounds and capnography. Before changing to lateral position, radial artery cannulation was preformed with difficulty. Anesthesia was maintained with 1 % sevoflurane, 4–6 mg/kg of propofol and 0.1–0.2 μg/kg/min of remifentanil. Guided by neuromuscular monitor, non-depolarization muscle reluxant atracurium was administered intermittently. Surgery lasted for 1 h and 40 min, with the blood loss of 300 ml. At the end of surgery and before he was transferred to post-anesthesia care unit (PACU), neuromuscular block was reversed and trachea was extubated. Intra-operative and postoperative course was uneventful.

## Discussion

Scleroderma is a multisystem disease that can affect every aspect of anesthesia especially airway management, which can pose a significant challenge for anesthesiologists. As a result, understanding its pathophysiological changes and implementing a meticulous pre-anesthesia check-up are essential when making an anesthetic plan. Central venous access is often necessitated due to the difficulty of a peripheral one because of skin thickening, flexion contractures and vasoconstriction. There are no specific contraindications for any type of anesthesia, however, selection still needs to be guided by analysis of organ dysfunction.

We chose general anesthesia for the patient, due to of positioning difficulty while he was awake and for the concern of prolonged duration of the local anesthetics (Bailey et al. 1999). In addition, because of some severe complications during regional anesthesia, patients may require endotracheal intubation which is always difficult and emergent. However, general anesthesia presents additional risk to scleroderma patients. Due to anatomical deformities caused by scleroderma, difficulties in mask ventilation and intubation should be considered and carefully prepared. As a result, an awake fiberoptic intubation may be the most appropriate technique; and in case of oral or pharyngeal hemorrhage or failed intubation, an awake tracheostomy should be ready to be performed (D’Angelo and Miller 1997).

In this case, we chose SOS to intubate our patient under rapid sequence introduction with non-depolarization muscle-relaxant succinylcholine. SOS conbines the benefits of lightwands and fiberoptic bronchoscope (FOB), which is recommended when the patient has limited mouth opening and neck motility. Maneuvering SOS around epiglottis, especially with jaw/tongue thrust, was easier and less time-consuming than maneuvering the FOB (Shukry et al. 2005). Besides, compared with FOB, SOS had a lower incidence of mucosal injury (Phua et al. 2012) as mucosal telangiectasia in sclerotic patients may lead to profuse bleeding so all procedures should be done carefully and gently. Furthermore, with its strong illumination, SOS can be used as a lightwand when blood or secretions obscure the fiberoptic view. For patients with scleroderma, orotracheal intubation is preferable to nasotracheal intubation as the fragility of the nasal mucosa increases the risk of severe nasal hemorrhage.

Esophageal dysmotility and lower esophageal sphincter incompetence may increase the risk of aspiration (D’Angelo and Miller 1997). In addition, Sellick’s manoeuvre may be ineffective due to esophageal fibrosis (Bansal and Hooda 2013). Administering antiemetic and an H_2_ blocker preoperatively should be taken into consideration as we used in our case. Nasogastric suctioning may also help to decrease the risk of aspiration. However, if there is a significant esophageal stricture, insertion may be difficult and should not be done forcefully because of possible esophageal perforation (Bansal and Hooda 2013).

Difficulty in measuring SpO_2_ with pulse oximetry might occur, and other techniques for SpO_2_ measurement need to be prepared and used. Pulse oximeter probes should be moved among digits during surgery to prevent ischemic damage (Roberts et al. 2002). Radial artery cannulation normally should be avoided because Raynaud’s phenomenon and even necrosis may occur (Thompson and Conklin 1983). But it may prove beneficial if the patient has severe cardiac disease and pulmonary hypertension.

Operating room temperature should be kept over 21 °C and intravenous fluids should be warmed before administration so as to minimize peripheral vasoconstriction (Roberts et al. 2002). On the other hand, overheating may also occur because of the hindered sweating caused by vasospasm, which for the worst presents as severe hyperthermia. Vasoconstrictor agents such as phenylephrine and dopamine should be avoided (Bansal and Hooda 2013). Eyes should be lubricated and taped to prevent corneal abrasions, because they have increased vulnerability to keratoconjuctivitis sicca and xerophthalmia. Attention also should be paid to special medication that patients take perioperatively. It is known that methyldopa and reserpine, which reduce norepinephrine levels centrally and peripherally, interact with anesthetic agents in a dose related fashion. Guanethidine reduces norepinephrine only in a peripheral way, which makes no change in MAC (minimum alveolar concentration) (Smoak 1982).

## Conclusion

Scleroderma is a multisystem disease that can pose a significant challenge for anesthetists. It is important to make a thorough evaluation preoperatively, so as to form a flexible anesthetic plan to minimize the morbidity and mortality. Airway management could be the most important and toughest problem during anesthesia. With a careful airway assessment, we assume that Shikani Optical Stylet could be reliable alternative to fiberoptic bronchoscope (FOB).

## References

[CR1] Bailey A, Wolmarans M, Rhodes S (1999). Spinal anaesthesia for caesarean section in a patient with systemic sclerosis. Anaesthesia.

[CR2] Bansal T, Hooda S (2013) Emergency surgery in a patient with scleroderma-anaesthetic challenges: a case report, Indian Anaesthetists’ Forum

[CR3] D’Angelo R, Miller R (1997). Pregnancy complicated by severe preeclampsia and thrombocytopenia in a patient with scleroderma. Anesth Analg.

[CR4] Phua DS, Mah CL, Wang CF (2012). The Shikani optical stylet as an alternative to the GlideScope (R) videolaryngoscope in simulated difficult intubations—a randomised controlled trial. Anaesthesia.

[CR5] Roberts JG, Sabar R, Gianoli JA, Kaye AD (2002). Progressive systemic sclerosis: clinical manifestations and anesthetic considerations. J Clin Anesth.

[CR6] Shukry M, Hanson RD, Koveleskie JR, Ramadhyani U (2005). Management of the difficult pediatric airway with Shikani Optical Stylet. Paediatr Anaesth.

[CR7] Smoak LR (1982). Anesthesia considerations for the patient with progressive systemic sclerosis (scleroderma). AANA J.

[CR8] Thompson J, Conklin KA (1983). Anesthetic management of a pregnant patient with scleroderma. Anesthesiology.

